# The Effect of Bariatric Surgery Volume on General Surgery Outcomes for Morbidly Obese Patients

**DOI:** 10.1155/2021/8945091

**Published:** 2021-10-31

**Authors:** Katheryn Hope Wilkinson, Ruizhe Wu, Aniko Szabo, Rana Higgins, Jon Gould, Tammy Kindel

**Affiliations:** ^1^Department of Surgery, Medical College of Wisconsin, 8900 W Doyne Avenue, Milwaukee, WI 53226, USA; ^2^Division of Biostatistics, Institute for Health and Equity, Medical College of Wisconsin, 8900 W Doyne Avenue, Milwaukee, WI 53226, USA

## Abstract

**Introduction:**

Bariatric surgery performed at high volume centers decreases length of stay, cost, and morbidity and mortality. The effect of a high volume of bariatric surgery procedures on outcomes may extend not just to bariatric surgery but to any general surgical procedure in morbidly obese patients. We hypothesized that patients with morbid obesity (body mass index >40 kg/m^2^) undergoing common, nonbariatric general surgery would have decreased morbidity and mortality at centers performing high volumes of bariatric surgery.

**Methods:**

The 2016 National Inpatient Sample (NIS) was used to identify the number of laparoscopic gastric bypass and sleeve gastrectomy performed at each hospital. Hospitals were classified as high volume bariatric hospitals (HVBH) ≥10 reported cases (50 actual)/year or low volume bariatric hospitals (LVBH) <10 reported cases (50 actual)/year, as NIS reports a 20% sample of actual cases. Patients with morbid obesity undergoing laparoscopic or open appendectomy, cholecystectomy, or ventral hernia repair were included for analysis. Propensity scores were developed based on available demographics, comorbidities, and hospital procedure volume. Postoperative complications during the index hospital admission, determined by ICD-10 code, were compared using inverse propensity weights. Differences were considered significant with a *p* value of <0.05.

**Results:**

The total number of general surgery patient cases analyzed was 14,028 from 2,482 hospitals, representing 70,140 admissions. The cohort of patients undergoing operations treated at HVBH were younger (*p*=0.03) with higher rates of COPD (*p*=0.04). Patients at LVBH had higher rates of nicotine dependence (*p*=0.0001) and obstructive sleep apnea (*p* < 0.001). On propensity-weighted analysis adjusting for preoperative comorbidities and hospital procedure volume, there were significantly higher rates of multiple postprocedure complications at LVBH, specifically, postprocedure respiratory failure for patients undergoing elective laparoscopic cholecystectomy, elective ventral hernia repair with mesh and appendectomy.

**Conclusion:**

Patients with morbid obesity may have an advantage in having general surgery procedures at HVBH. HVBH may have a volume-outcomes relationship where the hospital and staff familiarity with the management principles required to minimize the postoperative risk associated with morbid obesity and improve patient outcomes.

## 1. Introduction

Patients with morbid obesity (body mass index, BMI >40 kg/m^2^) have an increased risk of perioperative complications compared to normal weight individuals [1, 2]. These increased risks include wound complications, renal complications, venous thromboembolic (VTE) events, and pulmonary complications that vary by procedure [1–6].

Any strategy to reduce the increased risk of perioperative complications in morbidly obese patients is important, including the known benefit of VTE chemoprophylaxis, preoperative diagnosis and treatment of obstructive sleep apnea, and preoperative glycemic control. These pathways are a prominent part of perioperative patient care in accredited and high volume bariatric centers. Bariatric surgery performed at high volume centers decreases the length of stay, cost, and morbidity and mortality [7, 8]. The effect of a high volume of bariatric surgery procedures on outcomes may extend not just to bariatric surgery but to any general surgical procedure, as the hospital facility and staff are experienced in the surgical care of morbidly obese patients. We hypothesized that patients with morbid obesity undergoing common general surgery procedures would have decreased mortality and morbidity at centers performing high volumes of bariatric surgery.

## 2. Methods

### 2.1. Database

We used the 2016 National Inpatient Sample (NIS) to evaluate complication rates in patients with morbid obesity following traditionally, low-risk general surgery procedures. The NIS is a sample of 20% of discharges from all hospitals participating in the Healthcare Cost and Utilization Project. The NIS is unique in that patient information is linked to a unique hospital identifier.

### 2.2. Bariatric Surgery Volume

To estimate the number of bariatric surgeries performed by each hospital, the data was filtered for patients who underwent procedure codes of LRYGB (0D164ZA, 0D164ZB) and LSG (0DB64ZZ) with a diagnosis of morbid obesity (E66.01, E66.2). Patients were excluded if they had gastrointestinal neoplasm (C15–C26), inflammatory bowel disease (K50-51), or noninfectious colitis (K52). The number of cases for each hospital was attached to the NIS hospital number. Current metabolic and bariatric surgery quality and improvement program (MBSAQIP) center accreditation as a comprehensive center requires a program to perform ≥50 bariatric stapling surgeries per year. As the NIS data contains information for 20% of hospital discharges, we used a bariatric caseload of 10 or more as the threshold to define hospitals performing high volumes of bariatric surgery (HVBH) compared to low volume bariatric hospitals (LVBH).

### 2.3. Study Cohorts

We included patients with a diagnosis of morbid obesity (BMI > 40 kg/m^2^) who underwent nonelective or elective laparoscopic cholecystectomy (0FB40ZZ, 0FB44ZZ, 0FT44ZZ, 0FT40ZZ) and laparoscopic or open ventral hernia repair with (0WUF0JZ, 0WUF4JZ, 0WUF0KZ, 0WUF4KZ) or without mesh (0WQF0ZZ, 0WQF4ZZ). We included patients who underwent nonelective laparoscopic or open appendectomy (0DBJ0ZZ, 0DBJ4ZZ, 0DTJ0ZZ, 0DTJ4ZZ), and we excluded all appendectomies that were scheduled as elective due to inadequate procedure numbers for analysis.

### 2.4. Outcomes

Our primary outcomes of interest were postoperative complications, mortality, and length of stay. Complications were identified by ICD-10 codes (see Supplemental Table A) like methods previously described [9]. If a patient experienced one or more of the 24 complications, they were considered to have “any complication.”

### 2.5. Statistics

Descriptive statistics of preoperative demographics and comorbidities were compared with survey-weighted chi-squared tests adjusting for within-hospital clustering. Statistical analysis was performed using SAS version 9.4. Statistical significance was defined as *p* < 0.05.

### 2.6. Propensity Analysis

Propensity scores for the probability of having a procedure in a HVBH versus LVBH hospital were computed separately for each procedure and overall for the entire study population using the following predictors: age, median household income national quartile for patient ZIP Code, gender, renal failure, hypertension, chronic obstructive pulmonary disease, diabetes mellitus type 2, anemia, gastroesophageal reflux disease, chronic peptic ulcer disease, heart failure, hypothyroidism, history of VTE, history of pulmonary embolism (PE), nicotine dependence, obstructive sleep apnea, and the volume of the relevant procedure among all patients (i.e., the overall appendectomy volume when modeling the appendectomy population, etc.). Average treatment effect (ATE) weights were computed based on the propensity score, and weighted analysis was performed to estimate the effect of high bariatric volume versus low bariatric volume on the outcomes. Positive values imply higher rates in high bariatric volume hospitals. The propensity score weighted analysis was conducted with SAS version 9.4 PROC CAUSALTRT.

## 3. Results

### 3.1. All Procedures

The overall study cohort included 3,867 cases at HVBH and 10,161 cases at LVBH. The HVBH patients were younger (50.5 years vs. 51.2 years *p*=0.03), with a shorter length of stay (4.85 days vs. 5.39 days, *p* < 0.0001). Patients at HVBH also were more likely to be female and carry a preoperative diagnosis of hypertension, GERD, history of VTE or PE, nicotine dependence, and obstructive sleep apnea; see Table 1. Patients at LVBH were more likely to have preoperative renal failure. In unadjusted comparisons, postoperative rates of bowel obstruction (2.8% vs. 2.0%, *p*=0.01), pulmonary failure (2.9% vs. 1.6% *p* < 0.0001), and any complication (10.3% v. 7.76%, <0.0001) were higher at LVBH compared to HVBH. On propensity-weighted analysis, controlling for the entire set of preoperative comorbidities and the hospital volume differences, patients at LVBH were still more likely to have bowel obstructions (*p*=0.012), pulmonary failure (<0.0001), postoperative infection (0.005), wound disruption (*p*=0.03), or any postoperative complication (*p* < 0.0001).

### 3.2. Elective Laparoscopic Cholecystectomy

In the study cohort, 942 patients underwent elective laparoscopic cholecystectomy at HVBH (weighted *n* = 4,710) and 823 patients underwent elective laparoscopic cholecystectomy at LVBH (weighted *n* = 4,115). Patients at HVBH were younger (47.8 years vs. 51.0 years, *p* < 0.0001) and had shorter lengths of stay (3.13 days vs. 4.09 days, *p* < 0.0001); see Table 2. Patients had similar rates of many comorbidities; however, more patients at LVBH had COPD (7.86% vs. 5.22%, *p*=0.03) and heart failure (6.79% vs. 4.13%, *p*=0.01) and more patients at HBVH had anemia (1.94% vs. 0.75%, *p*=0.05) and OSA (42.9% vs. 33.7%, *p*=0.01). HVBH had higher levels of cholecystectomy volume (62.2/hospital vs. 40.4/hospital, *p* < 0.001). LVBH had higher rates of postoperative pulmonary failure (4.14% vs. 0.85%, *p* < 0.0001) and any complication (9.45% vs. 5.95%, *p*=0.01). On propensity-weighted analysis, patients at LVBH still had higher rates of pulmonary failure (*p* < 0.0001) any complication (*p*=0.04), and a significantly increased risk of postoperative mortality (*p*=0.05).

### 3.3. Nonelective Laparoscopic Cholecystectomy

In the study cohort, 1,047 patients underwent nonelective laparoscopic cholecystectomy at HVBH (weighted *n* = 5,235) and 4,399 patients underwent nonelective laparoscopic cholecystectomy at LVBH (weighted *n* = 21,995); see Table 3. Patients at LVBH had shorter lengths of stay (4.87 days vs. 5.25 days, *p*=0.05). Patients had similar rates of many comorbidities; however, more patients at HVBH had preoperative GERD and OSA (27.7% vs. 23.1%, *p*=0.003, and 23.4% vs. 19.5%, *p*=0.009, respectively). HVBH had higher levels of nonelective cholecystectomy volume (54.9/hospital vs. 39.0/hospital, *p* < 0.001). After propensity weighting, LVBH had higher rates of postprocedure hemorrhage (*p*=0.03) and any complication (*p*=0.05).

### 3.4. Elective Ventral Hernia Repair

For patients undergoing elective ventral hernia repair, there were 514 patients at HVBH (weighted *n* = 2,570) and 1,130 patients at LVBH (weighted *n* = 5,650). As shown in Table 4, patients at HVBH had significantly higher rates of preoperative GERD, peptic ulcer disease, history of pulmonary embolism, nicotine dependence, and OSA. HVBH had higher rates of ventral hernia repair volume with an average of 22 cases/hospital versus 13 cases/hospital (*p* < 0.0001). LVBH had higher rates of postoperative pulmonary failure (4.69% vs. 1.56%, *p*=0.002) and any complication (16.4% vs. 12.3%, *p*=0.03). After propensity weighting, patients at LVBH had still had higher rates of pulmonary failure (*p* < 0.0001) as well as having an increased risk of having any indiviudal complication (*p*=0.01).

### 3.5. Nonelective Ventral Hernia Repair

For patients undergoing nonelective ventral hernia repair, there were 314 patients at HVBH (weighted *n* = 1,570) and 1,143 patients at LVBH (weighted *n* = 5,715). As shown in Table 5, patients at HVBH had higher rates of history of pulmonary embolism, nicotine dependence, and OSA. HVBH had higher ventral hernia procedure volume with an average of 20.9 cases/hospital versus 10.8 cases/hospital (*p* < 0.0001). After propensity weighting, patients at LVBH had higher rates of postoperative death (*p*=0.03).

### 3.6. Nonelective Appendectomy

The patients that underwent appendectomy (nonelective cases only) included 205 patients at HVBH (weighted *n* = 1,025) and 870 patients at LVBH (weighted *n* = 4,350). Patients at LVBH had shorter length of stay (3.88 days vs. 4.7 days, *p*=0.02); see Table 6. There were no statistically significant differences in any preoperative comorbidity. There were more appendectomy cases done per hospital at HVBH (23.1/hospital vs. 17.0/hospital, *p* < 0.0001). LVBH had higher rates of postoperative pulmonary failure (3.22% vs. 0.49%, *p*=0.03) which persisted after propensity weighing (*p*=0.02).

## 4. Discussion

This analysis of the NIS 2016 dataset shows a higher rate of several complications for patients with morbid obesity having general surgery operations at hospitals performing low volumes of bariatric surgery. The complication most consistently increased across the studied groups was pulmonary failure, with higher rates in patients undergoing elective laparoscopic cholecystectomy, elective ventral hernia repair, and appendectomy. Despite adjusting for general surgery hospital procedure volume and preoperative comorbidities, we found a significantly higher rate of mortality at LVBH after elective laparoscopic cholecystectomy and nonelective ventral hernia repair.

Overall, the rate of several complications in NIS for morbidly obese patients is higher than reported in the literature for the general population. Prior reports of the rate of mortality following laparoscopic cholecystectomy are between 0 and 0.13%, compared to 0.23–0.96% in the NIS database [10]. Additionally, for laparoscopic cholecystectomy, the rate of any complication is reported as between 2.2% and 12% compared to 5.95 and 9.45% in patients with morbid obesity in the NIS database [10]. The rate of postoperative pulmonary failure in this current study (2.53%–4.53%) is similar to rates reported for patients undergoing bariatric surgery, with overall rates around 1.35% and the greatest rate of 4.1% after open gastric bypass [11]. This study highlights the overall increased complication rate for morbidly obese patients undergoing general surgery procedures.

The relationship between volumes and outcomes has been clearly observed but challenging at times to explain. Many studies have identified improved outcomes for patients undergoing surgery by surgeons who perform high volumes of complex surgery. Additionally, even when accounting for case mix, increased hospital case volume also was associated with improved outcomes independent of surgeon volume [12]. Specifically, for bariatric surgery, academic hospitals performing greater than 100 bariatric cases per year compared to those performing less than 50 per year had a shorter length of stay and fewer complications [7]. Bariatric surgery program site accreditation, which includes case-volume requirements, is associated with reduced morbidity and mortality [13,14]. Even among these accredited centers, the highest volume centers have the lowest rate of complications [15]. A systematic review of 24 papers on bariatric surgery volume and outcomes found both higher hospital volume and higher surgeon volume related to more positive outcomes [8]. The data in our study are unable to identify whether the surgeons performing the general surgery cases are also performing the bariatric surgeries or if it is other aspects of being a HVBH that improve general surgery outcomes [16].

We used bariatric volume thresholds in the NIS as a surrogate for accreditation, as site-specific data is not available in the NIS. Our hypothesis was that HVBH, likely to be accredited, follow very specific standards and regulations to improve the quality of care for patients with morbid obesity. These pathways often extend beyond technical aspects of the operation, to include facilities that have ICU equipment, chairs, beds, doorways, and toilets that support bariatric weights. Staff are required to have sensitivity training, training on safe patient transfer and mobilization, and education about signs and symptoms of postoperative complications unique to these patients. Accredited bariatric centers are also required to participate in quality improvement projects and data registries [17]. A prior study using the NIS database during a time when hospitals were not deidentified found a threefold reduction in hospital mortality at accredited bariatric centers [18]. As our results suggest, a bariatric volume to general surgery outcome relationship, it may be that the multimodality pathways required for accreditation at high volume bariatric centers create a culture of improved safety and outcomes for many general surgery procedures performed in morbidly obese patients.

The greatest source of increased morbidity for patients at LVBH was postprocedure respiratory failure. Risk factors for postprocedure respiratory failure include peripheral vascular disease, age >50, alcohol use, diabetes mellitus, smoking, obstructive sleep apnea, and chronic lung disease [11]. Patients with morbid obesity can have functional respiratory changes from surplus adipose tissue that causes reduced functional residual capacity and expiratory reserve volume [19]. In patients with morbid obesity, the nonhypoxic apnea time is reduced from three minutes in normal weight patients to one minute, and in the supine position, expiratory lung volume is reduced by 69% after the induction of anesthesia [20, 21]. Using a ramped or head elevated position for patients with morbid obesity has been shown to improve pulmonary compliance, allow for easier mask ventilation, and improve conditions for tracheal intubation by lengthening the time to desaturation by 50 seconds and increasing the likelihood of a grade one view by 30% [22, 23]. Additionally, patients are less likely to receive intraoperative lung protective ventilation with high volumes and low positive end-expiratory pressure [24]. This has been associated with higher rates of postprocedure pulmonary complications including unplanned need for oxygen (if not part of usual patient care), unexpected postoperative invasive or noninvasive mechanical ventilation, acute respiratory failure, acute respiratory distress syndrome, pneumonia, and pneumothorax. While the best ventilatory strategy for patients with morbid obesity is unknown, likely, anesthesia staff who are experienced with the management of morbidly obese patients result in a volume-outcome relationship for improved postoperative pulmonary outcomes [25].

The main limitations of this study are due to the nature of NIS collection. The NIS data is collected for analysis of costs and charges related to healthcare, not for quality purposes. Complication rates in NIS are higher than complications rates in NSQIP, attributed to the difference in data entry. For this study, it was necessary to use the NIS data because NSQIP and other databases do not associate the patients with a specific hospital, a feature of the NIS data that was crucial to perform this analysis. The ICD-10 codes are coded at hospital discharge and different states submit different code numbers to the NIS for collection in the data set. If a state only submits 10 codes, then both comorbid conditions and postoperative complications may be missed; however, this omission should be similar between the investigated cohorts. While it is widely believed that there is underreporting of obesity and other medical comorbidities in these databases, it is not clear which way would influence these results or if the large number of hospitals in each group would balance this confounding. Additionally, many cases of laparoscopic cholecystectomy, appendectomy, and ventral hernia repair may be conducted as outpatient surgery and are not included in this analysis. This means that included patients likely represent a sicker cohort than the overall cohort of patients seen at each set of hospitals. And lastly, a significant limitation of the NIS is that NIS approximates a 20-percent stratified sample of discharges from hospitals rather than a true census of discharges, so the categorization of hospital volume could be flawed and should and will be verified in future studies by databases that can provide a precise census of discharges from individual hospitals, such as individual state data through the HCUP Central Distributor. While these limitations are significant, we believe the outcome differences seen between the two groups are robust enough that the importance should not be dismissed. These descriptive results are compelling that there is room for improvement in the care of the general surgery patient with morbid obesity.

## 5. Conclusions

Patients with morbid obesity may have an advantage in having general surgery procedures performed at HVBH. HVBH may have a volume-outcomes relationship where the hospital and staff familiarity with the management principles required to minimize the postoperative risk associated with morbid obesity improve patient outcomes for general surgery procedures.

## Figures and Tables

**Table 1:** Preoperative comorbidities and postoperative complications of patients with morbid obesity undergoing cholecystectomy, appendectomy, or ventral hernia repair.

Preoperative variables	HVBH *n* = 3,867 Weighted *n* = 19,335	LVBH *n* = 10,161 Weighted *n* = 50,805	*p* value unadjusted		
*Age (years, mean* *±* *SE)*	50.5 ± 0.3	51.2 ± 0.2	**0.03**		
*Length of stay (days, mean* *±* *SE)*	4.9 ± 0.1	5.4 ± 0.1	**<0.0001**		
*Median household income quartile for zip* code	2.32 ± 0.04	2.25 ± 0.02	**0.0005**		
*Gender, female (% ± SE)*	72.7 ± 0.8	69.4 ± 0.5	**0.0003**		
*Renal failure (% ± SE)*	1.1 ± 0.2	1.5 ± 0.1	**0.08**		
*Hypertension (% ± SE)*	52.4 ± 0.9	49.3 ± 0.5	**0.002**		
*Chronic obstructive pulmonary sis. (% ± SE)*	8.9 ± 0.5	10.3 ± 0.3	**0.03**		
*Diabetes mellitus type 2 (% ± SE)*	10.2 ± 0.6	9.8 ± 0.3	0.51		
*Anemia (% ± SE)*	0.78 ± 0.18	0.53 ± 0.07	0.15		
*Gastroesophageal reflux disease (% ± SE)*	33.2 ± 1.0	24.9 ± 0.5	**<0.0001**		
*Chronic peptic ulcer disease (% ± SE)*	0.72 ± 0.15	0.82 ± 0.09	0.61		
*Heart failure (% ± SE)*	7.5 ± 0.5	8.4 ± 0.3	0.10		
*Hypothyroidism (% ± SE)*	12.7 ± 0.5	12.7 ± 0.3	0.97		
*History of venous thromboembolism (% ± SE)*	4.1 ± 0.4	3.1 ± 0.2	**0.005**		
*History of pulmonary embolism (% ± SE)*	3.4 ± 0.3	2.1 ± 0.1	**<0.0001**		
*Nicotine dependence (% ± SE)*	20.1 ± 0.9	16.6 ± 0.4	**0.0001**		
*Obstructive sleep apnea (% ± SE)*	35.2 ± 1.3	24.8 ± 0.5	**<0.0001**		

Postprocedure complications	HVBH	LVBH	*p* value unadjusted	ATE (95% CI)	*p* value adjusted
*Cerebrovascular infarct (% ± SE)*	0.03 ± 0.03	0.03 ± 0.02	0.91	—	—
*Shock (% ± SE)*	0.39 ± 0.12	0.29 ± 0.05	0.39	—	—
*Hemorrhage or hematoma (% ± SE)*	0.18 ± 0.07	0.18 ± 0.04	0.96	—	—
*Bowel obstruction (% ± SE)*	1.99 ± 0.26	2.82 ± 0.18	**0.01**	−0.76 (−1.36/0.16)	**0.01**
*Enterotomy (% ± SE)*	0.57 ± 0.13	0.67 ± 0.08	0.53	—	—
*GI bleed (% ± SE)*	0.41 ± 0.10	0.53 ± 0.07	0.36	−0.19 (−0.44/0.06)	0.13
*Pulmonary insufficiency (% ± SE)*	0.31 ± 0.10	0.2 ± 0.05	0.25	—	—
*Pulmonary failure (% ± SE)*	1.55 ± 0.21	2.9 ± 0.18	**<0.0001**	−1.31 (−1.93/−0.69)	**<0.0001**
*Pneumonia (% ± SE)*	0.41 ± 0.11	0.5 ± 0.07	0.51	—	—
*Genitourinary complication (% ± SE)*	0.16 ± 0.06	0.14 ± 0.04	0.81	—	—
*Postoperative infection (% ± SE)*	1.14 ± 0.19	1.46 ± 0.11	0.18	−0.59 (−1.01/−0.18)	**0.005**
*Wound complication (% ± SE)*	0.16 ± 0.06	0.12 ± 0.03	0.58	−0.42 (−0.80/−0.03)	**0.03**
*Venous thromboembolism (% ± SE)*	0.31 ± 0.09	0.45 ± 0.07	0.24	—	—
*Pulmonary embolism* (*% ± SE*)	0.65 ± 0.14	0.54 ± 0.07	0.49	—	—
*“Other” complications* (*% ± SE*)	0.18 ± 0.07	0.27 ± 0.05	0.36	—	—
*Death* (*% ± SE*)	0.59 ± 0.12	0.8 ± 0.09	0.19	−0.10 (−0.47/0.27)	0.60
*Patient had any complication* (*% ± SE*)	7.8 ± 0.5	10.3 ± 0.3	**<0.0001**	−3.32 (−4.43/−2.21)	**<0.0001**

HVBH: high volume bariatric hospital; LVBH: low volume bariatric hospital; ATE: average treatment effect.

**Table 2:** Preoperative comorbidities and postoperative complications of patients with morbid obesity undergoing elective laparoscopic cholecystectomy.

Preoperative variables	HVBH *n* = 942 Weighted *n* = 4710	LVBH *n* = 823 Weighted *n* = 4115	*p* value unadjusted		
*Age (years, mean* *±* *SE)*	47.9 ± 0.6	51.0 ± 0.5	**<0.0001**		
*Length of stay (days, mean* *±* *SE)*	3.3 ± .02	4.1 ± 0.2	**<0.0001**		
*Median household income quartile for zip* code	2.5 ± 0.05	2.4 ± 0.04	**0.05**		
*Gender, female (% ± SE)*	75.5 ± 1.5	71.5 ± 1.4	0.06		
*Renal failure (% ± SE)*	0.73 ± 0.30	1.17 ± 0.33	0.34		
*Hypertension (% ± SE)*	53.3 ± 1.6	52.4 ± 1.5	0.68		
*Chronic obstructive pulmonary dis. (%± SE)*	5.2 ± 0.8	7.9 ± 0.9	**0.03**		
*Diabetes mellitus type 2 (%± SE)*	9.5 ± 1.0	11.7 ± 1.0	0.12		
*Anemia (%± SE)*	1.94 ± 0.71	0.74 ± 0.27	**0.05**		
*Gastroesophageal reflux disease (%± SE)*	39.5 ± 2.1	36.9 ± 1.9	0.38		
*Chronic peptic ulcer disease (%± SE)*	0.49 ± 0.30	0.74 ± 0.26	0.54		
*Heart failure (%± SE)*	4.1 ± 0.7	6.8 ± 0.8	**0.01**		
*Hypothyroidism (%± SE)*	11.5 ± 1.0	12.5 ± 1.0	0.49		
*History of venous thromboembolism (%± SE)*	3.7 ± 0.8	2.6 ± 0.5	0.19		
*History of pulmonary embolism (%± SE)*	2.3 ± 0.5	1.6 ± 0.4	0.25		
*Nicotine dependence (%± SE)*	21.4 ± 2.1	19.0 ± 1.3	0.30		
*Obstructive sleep apnea (%± SE)*	42.9 ± 3.6	33.7 ± 1.6	**0.01**		
*Cholecystectomy volume (cases/hospital ± SE)*	62.2 ± 8.1	40.4 ± 1.2	**<0.00**		

Postprocedure complications	HVBH	LVBH	*p* value unadjusted	ATE (95% CI)	*p* value adjusted
*Shock (% ± SE)*	0.24 ± 0.17	0.42 ± 0.21	0.52	—	—
*Hemorrhage or hematoma (% ± SE)*	0.49 ± 0.22	0.42 ± 0.21	0.84	—	—
*Bowel obstruction (%± SE)*	1.22 ± 0.39	1.91 ± 0.45	0.25	−0.50 (−1.57/0.58)	0.36
*Enterotomy (%± SE)*	0.73 ± 0.30	0.64 ± 0.24	0.81	0.37 (−0.35/1.10)	0.32
*GI bleed (%± SE)*	0.49 ± 0.25	0.85 ± 0.28	0.34	−0.33 (−1.10/0.43)	0.39
*Pulmonary insufficiency (%± SE)*	0.12 ± 0.12	0.42 ± 0.18	0.22	—	—
*Pulmonary failure (%± SE)*	0.85 ± 0.30	4.14 ± 0.67	**<0.0001**	−3.27 (−4.8/−1.73)	**<0.0001**
*Pneumonia (%± SE)*	0.61 ± 0.28	0.42 ± 0.18	0.57	—	—
*Genitourinary complication (%± SE)*	0.24 ± 0.17	0.21 ± 0.15	0.89	—	—
*Postoperative infection (%± SE)*	0.85 ± 0.31	0.85 ± 0.30	1.00	0.18 (−0.90/1.26)	0.74
*Wound complication (%± SE)*	0.12 ± 0.12	0.11 ± 0.11	0.92	—	—
*Venous thromboembolism (%± SE)*	0.12 ± 0.12	0.42 ± 0.21	0.23	—	—
*Pulmonary embolism* (%*± SE*)	0.36 ± 0.21	0.32 ± 0.18	0.87	—	—
*“Other” complications* (%*± SE*)	0.12 ± 0.12	0.11 ± 0.11	0.92	—	—
*death* (%*± SE*)	0.24 ± 0.17	0.96 ± 0.32	0.06	−0.65 (−1.30/0.01)	**0.05**
*Patient had any complication* (%*± SE*)	6.0 ± 0.9	9.5 ± 1.0	**0.01**	−2.67 (−5.25/−0.09)	**0.04**

HVBH: high volume bariatric hospital; LVBH: low volume bariatric hospital; ATE: average treatment effect.

**Table 3:** Preoperative comorbidities and postoperative complications of patients with morbid obesity undergoing nonelective laparoscopic cholecystectomy.

Preoperative variables	HVBH *n* = 1047 Weighted *n* = 5235	LVBH *n* = 4399 Weighted *n* = 21995	*p* value unadjusted		
*Age (years, mean* *±* *SE)*	48.1 ± 0.6	48.5 ± 0.3	0.47		
*Length of stay (days, mean* *±* *SE)*	5.3 ± 0.2	4.9 ± 0.1	**0.05**		
*Median household income quartile for zip* code	2.2 ± 0.1	2.2 ± 0.0	0.49		
*Gender, female (%± SE)*	27.4 ± 1.4	30.0 ± 0.7	0.10		
*Renal failure (%± SE)*	1.8 ± 0.4	1.6 ± 0.2	0.58		
*Hypertension (%± SE)*	45.5 ± 1.6	44.6 ± 0.8	0.65		
*Chronic obstructive pulmonary dis. (%± SE)*	8.2 ± 0.9	9.0 ± 0.4	0.48		
*Diabetes mellitus type 2 (%± SE)*	10.4 ± 0.9	8.9 ± 0.4	0.12		
*Anemia (%± SE)*	0.67 ± 0.25	0.36 ± 0.09	0.17		
*Gastroesophageal reflux disease (%± SE)*	27.7 ± 1.5	23.1 ± 0.7	**0.003**		
*Chronic peptic ulcer disease (%± SE)*	0.76 ± 0.27	1.18 ± 0.16	0.24		
*Heart failure (%± SE)*	10.0 ± 1.0	8.2 ± 0.4	0.08		
*Hypothyroidism (%± SE)*	10.7 ± 1.0	11.6 ± 0.5	0.43		
*History of venous thromboembolism (%± SE)*	3.2 ± 0.6	2.7 ± 0.2	0.42		
*History of pulmonary embolism (%± SE)*	2.1 ± 0.4	1.7 ± 0.2	0.36		
*Nicotine dependence (%± SE)*	17.1 ± 1.2	15.7 ± 0.6	0.31		
*Obstructive sleep apnea (%± SE)*	23.4 ± 1.4	19.5 ± 0.6	**0.009**		
*Cholecystectomy volume (cases/hospital ± SE)*	54.9 ± 2.5	39.0 ± 0.9	**<0.0001**		

Postprocedure complications	HVBH	LVBH	*p* value unadjusted	ATE (95% CI)	*p* value adjusted
*Shock (%± SE)*	0.19 ± 0.13	0.11 ± 0.05	0.53	—	—
*Hemorrhage or hematoma (%± SE)*	0.10 ± 0.10	0.27 ± 0.08	0.29	−0.20 (−0.39/−0.02)	**0.03**
*Cardiac arrest (%± SE)*	0.19 ± 0.13	0.05 ± 0.03	0.12	−0.49 (−1.01/0.03)	0.07
*Bowel obstruction (%± SE)*	0.86 ± 0.28	1.14 ± 0.16	0.43	−0.19 (−0.55/0.17)	0.31
*Enterotomy (%± SE)*	0.38 ± 0.19	0.48 ± 0.10	0.68	−0.17 (−0.53/0.19)	0.36
*GI bleed (%± SE)*	0.19 ± 0.14	0.39 ± 0.09	0.33	—	—
*Pulmonary failure (%± SE)*	1.2 ± 0.3	1.6 ± 0.2	0.33	−0.43 (−1.23/0.37)	0.29
*Pneumonia (%± SE)*	0.19 ± 0.13	0.25 ± 0.07	0.72	−0.15 (−0.39/0.08)	0.20
*Genitourinary complication (%± SE)*	0.19 ± 0.13	0.09 ± 0.04	0.38	—	—
*Postoperative infection (%± SE)*	0.29 ± 0.16	0.43 ± 0.10	0.50	−0.24 (−0.55/0.08)	0.14
*Wound disruption (%± SE)*	0.29 ± 0.16	0.27 ± 0.08	0.94	−0.17 (−0.41/0.06)	0.15
*Venous thromboembolism (%± SE)*	0.10 ± 0.10	0.25 ± 0.07	0.34	−0.17 (−0.41/0.07)	0.17
*Pulmonary embolism* (%*± SE*)	0.29 ± 0.16	0.30 ± 0.08	0.96	0.09 (−0.38/0.56)	0.70
*“Other” complications* (%*± SE*)	0.10 ± 0.10	0.16 ± 0.06	0.63	—	—
*Death* (%*± SE*)	0.67 ± 0.25	0.41 ± 0.10	0.26	0.22 (−0.27/0.71)	0.39
*Patient had any complication* (%*± SE*)	3.82 ± 0.58	5.11 ± 0.34	0.08	−1.88 (−3.17/−0.58)	**0.005**

HVBH: high volume bariatric hospital; LVBH: low volume bariatric hospital; ATE: average treatment effect.

**Table 4:** Preoperative comorbidities and postoperative complications of patients with morbid obesity undergoing elective ventral hernia repair.

Preoperative variables	HVBH *n* = 514 Weighted *n* = 2570	LVBH *n* = 1130 Weighted *n* = 5650	*p* value unadjusted		
*Age (years, mean* *±* *SE)*	54.8 ± 0.5	55.89 ± 0.4	0.08		
*Length of stay (days, mean* *±* *SE)*	5.1 ± 0.3	5.8 ± 0.3	0.17		
*Median household income quartile for zip* code	2.3 ± 0.05	2.3 ± 0.03	0.80		
*Gender, female (%± SE)*	72.2 ± 2.2	71.7 ± 1.4	0.87		
*Renal failure (%± SE)*	0.39 ± 0.28	1.06 ± 0.30	0.17		
*Hypertension (%± SE)*	57.4 ± 2.1	55.3 ± 1.5	0.42		
*Chronic obstructive pulmonary dis. (%± SE)*	13.0 ± 1.4	13.5 ± 1.0	0.77		
*Diabetes mellitus type 2 (%± SE)*	10.9 ± 1.4	11.2 ± 1.0	0.88		
*Anemia (%± SE)*	0.58 ± 0.34	0.97 ± 0.28	0.42		
*Gastroesophageal reflux disease (%± SE)*	35.2 ± 2.3	29.6 ± 1.4	**0.03**		
*Chronic peptic ulcer disease (%± SE)*	1.56 ± 0.59	0.44 ± 0.20	**0.02**		
*Heart failure (%± SE)*	5.8 ± 1.0	6.2 ± 0.7	0.78		
*Hypothyroidism (%± SE)*	15.0 ± 1.5	14.7 ± 1.0	0.87		
*History of venous thromboembolism (%± SE)*	6.4 ± 1.1	4.3 ± 0.6	0.07		
*History of pulmonary embolism (%± SE)*	6.4 ± 1.0	2.7 ± 0.5	**0.0003**		
*Nicotine dependence (%± SE)*	25.7 ± 2.0	18.9 ± 1.1	**0.002**		
*Obstructive sleep apnea (%± SE)*	38.5 ± 2.2	30.1 ± 1.3	**0.001**		
*Ventral hernia volume (cases/hospital ± SE)*	22.4 ± 2.2	13.2 ± 0.6	**<0.0001**		

Postprocedure complications	HVBH	LVBH	*p* value unadjusted	ATE (95% CI)	*p* value adjusted
*Shock (%± SE)*	0.97 ± 0.63	0.27 ± 0.15	0.11	—	—
*Bowel obstruction (%± SE)*	3.3 ± 0.9	4.5 ± 0.6	0.28	−1.08 (−3.03/0.88)	0.30
*Enterotomy (%± SE)*	1.17 ± 0.46	0.97 ± 0.28	0.71	0.12 (−0.93/1.17)	0.82
*GI bleed (%± SE)*	0.39 ± 0.27	0.53 ± 0.20	0.69	—	—
*Pulmonary insufficiency (%± SE)*	0.78 ± 0.38	0.27 ± 0.15	0.13	—	—
*Pulmonary failure (%± SE)*	1.6 ± 0.5	4.7 ± 0.7	**0.002**	−3.80 (−5.55/−2.04)	**<0.0001**
*Pneumonia (%± SE)*	0.78 ± 0.39	0.97 ± 0.29	0.70	0.01 (−1.06/1.07)	0.99
*Genitourinary complication (%± SE)*	0.19 ± 0.19	0.27 ± 0.15	0.79	—	—
*Postoperative infection (%± SE)*	1.8 ± 0.6	2.5 ± 0.5	0.37	−0.71 (−2.48/1.07)	0.44
*Wound complication (%± SE)*	0.58 ± 0.27	0.35 ± 0.18	0.45	−0.35 (−1.84/1.14)	0.64
*Venous thromboembolism (%± SE)*	0.78 ± 0.38	0.44 ± 0.20	0.39	—	—
*Pulmonary embolism* (%*± SE*)	1.17 ± 0.55	0.35 ± 0.17	0.06	0.77 (−0.47/2.00)	0.22
*“Other” complications* (%*± SE*)	0.19 ± 0.19	0.44 ± 0.20	0.44	−0.39 (−1.12/0.34)	0.29
*Death* (%*± SE*)	0.58 ± 0.34	0.89 ± 0.28	0.52	−4.91 (−8.68/−1.15)	**0.01**
*Patient had any complication* (%*± SE*)	12.3 ± 1.5	16.4 ± 1.1	**0.03**	—	—

HVBH: high volume bariatric hospital; LVBH: low volume bariatric hospital; ATE: average treatment effect.

**Table 5:** Preoperative comorbidities and postoperative complications of patients with morbid obesity undergoing nonelective ventral hernia repair.

Preoperative variables	HVBH *n* = 314 Weighted *n* = 1570	LVBH *n* = 1143 Weighted *n* = 5715	*p* value unadjusted		
*Age (years, mean* *±* *SE)*	55.5 ± 0.7	56.4 ± 0.4	0.27		
*Length of stay (days, mean)*	8.0 ± 0.6	7.7 ± 0.3	0.56		
*Median household income quartile for zip* code	2.2 ± 0.1	2.2 ± 0.0	0.96		
*Gender, female (%± SE)*	74.5 ± 2.4	70.1 ± 1.3	0.12		
*Renal failure (%± SE)*	0.96 ± 0.6	1.4 ± 0.4	0.54		
*Hypertension (%± SE)*	57.3 ± 3.0	58.4 ± 1.4	0.75		
*Chronic obstructive pulmonary dis. (%± SE)*	17.8 ± 2.2	15.3 ± 1.0	0.29		
*Diabetes mellitus type 2 (%± SE)*	12.7 ± 1.9	11.7 ± 0.9	0.63		
*Anemia (%± SE)*	0.32 ± 0.32	0.79 ± 0.26	0.37		
*Gastroesophageal reflux disease (%± SE)*	27.4 ± 2.6	22.9 ± 1.2	0.11		
*Chronic peptic ulcer disease (%± SE)*	0.96 ± 0.55	0.61 ± 0.23	0.51		
*Heart failure (%± SE)*	13.4 ± 2.0	12.7 ± 1.0	0.75		
*Hypothyroidism (%± SE)*	14.6 ± 2.0	15.5 ± 1.1	0.72		
*History of venous thromboembolism (%± SE)*	5.7 ± 1.2	4.8 ± 0.6	0.48		
*History of pulmonary embolism (%± SE)*	5.7 ± 1.4	3.1 ± 0.5	**0.03**		
*Nicotine dependence (%± SE)*	21.7 ± 2.5	16.1 ± 1.1	**0.03**		
*Obstructive sleep apnea (%± SE)*	35.7 ± 2.7	30.1 ± 1.1	0.06		
*Ventral hernia volume (cases/hospital ± SE)*	20.9 ± 1.8	10.8 ± 0.4	**<0.0001**		

Postprocedure complications	HVBH	LVBH	*p* value unadjusted	ATE (95% CI)	*p* value adjusted
*Shock (%± SE)*	0.64 ± 0.32	0.44 ± 0.17	0.56	—	—
*Cardiac arrest (%± SE)*	0.32 ± 0.32	0.26 ± 0.15	0.87	—	—
*Bowel obstruction (%± SE)*	4.1 ± 1.2	5.5 ± 0.7	0.35	−0.79 (−3.80/2.23)	0.61
*Enterotomy (%± SE)*	0.32 ± 0.32	0.79 ± 0.26	0.37	−0.45 (−1.21/0.31)	0.24
*GI bleed (%± SE)*	0.96 ± 0.55	0.96 ± 0.29	0.99	−0.41 (−1.33/0.52)	0.39
*Pulmonary insufficiency (%± SE)*	0.64 ± 0.45	0.26 ± 0.15	0.31	—	—
*Pulmonary failure (%± SE)*	4.1 ± 1.1	4.6 ± 0.6	0.70	−0.08 (−2.81/2.66)	0.96
*Pneumonia (%± SE)*	0.96 ± 0.55	0.61 ± 0.23	0.52	0.92 (−0.83/2.67)	0.30
*Postoperative infection (%± SE)*	2.6 ± 0.9	3.3 ± 0.5	0.47	−1.72 (−3.49/0.06)	0.06
*Wound complication (%± SE)*	0.32 ± 0.32	0.09 ± 0.09	0.33	−0.83 (−2.94/1.27)	0.44
*Venous thromboembolism (%± SE)*	1.59 ± 0.70	0.96 ± 0.27	0.33	0.15 (−1.04/1.35)	0.80
*Pulmonary embolism* (%*± SE*)	1.9 ± 0.8	1.5 ± 0.4	0.59	−0.50 (−1.74/0.75)	0.43
*Death* (%*± SE*)	0.32 ± 0.32	1.66 ± 0.37	0.07	−1.15 (−2.18/−0.12)	**0.03**
*Patient had any complication* (%*± SE*)	17.8 ± 2.1	17.8 ± 1.1	0.98	−2.49 (−7.36/2.39)	0.32

HVBH: high volume bariatric hospital; LVBH: low volume bariatric hospital; ATE: average treatment effect.

**Table 6:** Preoperative comorbidities and postoperative complications of patients with morbid obesity undergoing nonelective appendectomy.

Preoperative variables	HVBH *n* = 205 Weighted *n* = 1025	LVBH *n* = 870 Weighted *n* = 4350	*p* value unadjusted		
*Age (years, mean* *±* *SE)*	45.6 ± 1.3	44.4 ± 0.6	0.34		
*Length of stay (days, mean* *±* *SE)*	4.8 ± 0.6	3.9 ± 0.1	**0.02**		
*Median household income quartile for zip* code	2.2 ± 0.1	2.3 ± 0.0	**0.05**		
*Gender, female (%± SE)*	40.5 ± 3.5	40.1 ± 1.6	0.92		
*Renal failure (%± SE)*	1.5 ± 0.8	1.4 ± 0.4	0.93		
*Hypertension (%± SE)*	42.0 ± 3.6	41.0 ± 1.6	0.82		
*Chronic obstructive pulmonary dis. (%± SE)*	8.3 ± 2.0	7.2 ± 0.9	0.61		
*Diabetes mellitus type 2 (%± SE)*	9.8 ± 2.1	9.5 ± 1.0	0.92		
*Gastroesophageal reflux disease (%± SE)*	18.0 ± 2.7	15.5 ± 1.2	0.37		
*Heart failure (%± SE)*	3.9 ± 1.2	5.8 ± 0.8	0.26		
*Hypothyroidism (%± SE)*	11.7 ± 2.2	8.7 ± 0.9	0.19		
*History of venous thromboembolism (%± SE)*	1.5 ± 0.8	1.3 ± 0.4	0.82		
*History of pulmonary embolism (%± SE)*	1.0 ± 0.7	1.2 ± 0.4	0.83		
*Nicotine dependence (%± SE)*	17.1 ± 2.8	13.2 ± 1.1	0.16		
*Obstructive sleep apnea (%± SE)*	22.9 ± 3.0	19.7 ± 1.4	0.30		
*Appendectomy volume (cases/hospital ± SE)*	23.1 ± 1.4	17.0 ± 0.5	**<0.0001**		

Postprocedure complications	HVBH	LVBH	*p* value unadjusted	ATE (95% CI)	*p* value adjusted
*Bowel obstruction (%± SE)*	3.4 ± 1.3	4.8 ± 0.7	0.38	−1.53 (−4.60/1.55)	0.33
*Pulmonary insufficiency (%± SE)*	0.49 ± 0.49	0.11 ± 0.11	0.26	—	—
*Pulmonary failure (%± SE)*	0.49 ± 0.49	3.22 ± 0.59	**0.03**	−2.28 (−4.20/−0.35)	**0.02**
*Pneumonia (%± SE)*	0.49 ± 0.48	0.57 ± 0.26	0.88	—	—
*Postoperative infection (%± SE)*	0.98 ± 0.67	0.69 ± 0.28	0.66	—	—
*Venous thromboembolism (%± SE)*	0.49 ± 0.49	0.23 ± 0.16	0.53	—	—
*Pulmonary embolism* (%*± SE*)	0.49 ± 0.49	0.46 ± 0.23	0.96	—	—
*Death* (%*± SE*)	0.98 ± 0.63	0.23 ± 0.16	0.10	—	—
*Patient had any complication* (%*± SE*)	6.8 ± 2.0	9.4 ± 1.0	0.28	−3.29 (−7.23/0.64)	0.10

HVBH: high volume bariatric hospital; LVBH: low volume bariatric hospital; ATE: average treatment effect.

## Data Availability

The data are available from the Healthcare Cost and Utilization Project: https://www.hcup-us.ahrq.gov/db/nation/nis/nisdbdocumentation.jsp.

## Supplementary Materials

Supplementary Materials Table A: ICD 10 codes for postoperative complications.
