# Supplementary material for: The Effect of Bariatric Surgery Volume on General Surgery Outcomes for Morbidly Obese Patients
Source: J Obes. 2021 Oct 31;2021:8945091. doi: 10.1155/2021/8945091 (PMC8572633; doi:10.1155/2021/8945091)
Supplement: Supplementary Materials — Table A: ICD 10 codes for postoperative complications. [file 8945091.f1.docx]

**Supplemental Material Table A**

Table A: ICD 10 codes for post-operative complications

| Post-Op Shock | T81.10, T81.11, T81.12, T81.19 |
| --- | --- |
| Wound disruption | T81.3, T81.30, T81.31, T81.32 |
| Post-Operative Infections | T81.40, T81.40, T81.42, T81.43, T81.44, T81.49 |
| Failed intubation | T88.4 |
| Other complications from surgical or medical care | T81.8, T88.8, T88.9 |
| Post-procedure obstruction | K91.3, K91.30, K91.31, K91.32 |
| Post-cholecystectomy syndrome | K91.5 |
| Intra-operative hemorrhage and hematoma | K91.61, K91.62 |
| Enterotomy | K91.7, K91.71, K91.72 |
| Post-operative hepatic failure | K91.82 |
| Post-operative hepatorenal syndrome | K91.83 |
| Post-operative GI bleed | K91.84, K91.840, K91.841 |
| Post-operative pulmonary insufficiency | J95.2, J95.3 |
| Post-operative respiratory failure | J95.82, J95.821, J95.821 |
| Post-operative pneumonia | J95.89 |
| Post procedure cardiac insufficiency | I97.111 |
| Peri-procedure cardiac arrest | I97.121, I97.711 |
| Post procedure heart failure | I97.131 |
| Post-operative hemorrhage, hematoma or seroma | I97.62, I97.620, I97.621, I97.622 |
| Peri-operative cerebrovascular infarction | I97.811, I97.821 |
| Wound complications | L76.02, L76.12, L76.32, L76.34, L76.81, L76.82 |
| Venous thromboembolism | I82.4, I82.40, I82.401, I82.402, I82.403, I82.409, I82.41, I82.411, I82.412, I82.419, I82.42, I82.421, I82.422, I82.423, I82.429, I82.43, I82.431, I82.432, I82.433, I82.439, I82.44, I82.441, I82.442, I82.443, I82.449, I82.49, I82.4912, I82.493, I82.499, I82.4Y, I82.4Y2, I82.4Y3, I82.4Y9, I82.4Z, I82.4Z1, I82.4Z2, I82.4Z3, I82.4Z9 |
| Pulmonary Embolism | I26.0, I26.01, I26.02, I26.09, I26.90, I26.92, I26.99 |
| Post procedural (acute) (chronic) kidney failure | N99.0 |
